# Plastic ingestion by juvenile polar cod (*Boreogadus saida*) in the Arctic Ocean

**DOI:** 10.1007/s00300-018-2283-8

**Published:** 2018-02-20

**Authors:** Susanne Kühn, Fokje L. Schaafsma, Bernike van Werven, Hauke Flores, Melanie Bergmann, Marion Egelkraut-Holtus, Mine B. Tekman, Jan A. van Franeker

**Affiliations:** 1 Wageningen Marine Research, Ankerpark 27, 1781 AG Den Helder, The Netherlands; 2 University of Utrecht, Heidelberglaan 2, 3584 CS Utrecht, The Netherlands; 3 Alfred-Wegener-Institut, Helmholtz-Zentrum für Polar- und Meeresforschung, Am Handelshafen 12, 27570 Bremerhaven, Germany; 4 Shimadzu Europa GmbH, Albert-Hahn-Str. 6-10, 47269 Duisburg, Germany

**Keywords:** Polar cod (*Boreogadus saida*), Microplastic, Arctic, Airborne micro-fibre contamination

## Abstract

One of the recently recognised stressors in Arctic ecosystems concerns plastic litter. In this study, juvenile polar cod (*Boreogadus saida*) were investigated for the presence of plastics in their stomachs. Polar cod is considered a key species in the Arctic ecosystem. The fish were collected both directly from underneath the sea ice in the Eurasian Basin and in open waters around Svalbard. We analysed the stomachs of 72 individuals under a stereo microscope. Two stomachs contained non-fibrous microplastic particles. According to µFTIR analysis, the particles consisted of epoxy resin and a mix of Kaolin with polymethylmethacrylate (PMMA). Fibrous objects were excluded from this analysis to avoid bias due to contamination with airborne micro-fibres. A systematic investigation of the risk for secondary micro-fibre contamination during analytical procedures showed that precautionary measures in all procedural steps are critical. Based on the two non-fibrous objects found in polar cod stomachs, our results show that ingestion of microplastic particles by this ecologically important fish species is possible. With increasing human activity, plastic ingestion may act as an increasing stressor on polar cod in combination with ocean warming and sea-ice decline in peripheral regions of the Arctic Ocean. To fully assess the significance of this stressor and its spatial and temporal variability, future studies must apply a rigorous approach to avoid secondary pollution.

**Electronic supplementary material:**

The online version of this article (10.1007/s00300-018-2283-8) contains supplementary material, which is available to authorised users.

## Introduction

Debris ingestion by a wide range of marine organisms has been demonstrated in various studies from all over the world. At least 331 marine species have been documented to ingest plastic between the 1960s and 2015 of which 92 were fish species (Kühn et al. 2015). However, this is a rapidly developing field and the online database ‘Litterbase’ (Bergmann et al. 2017b) currently holds 168 fish species. The commercial value and worldwide consumption of fish have triggered an interest to study the abundance of plastic in fish, as it raises concerns about human exposure (Rochman et al. 2015).

Marine debris and in particular plastics have been found in all ocean basins of the world (Barnes et al. 2009; Eriksen et al. 2014; Galgani et al. 2015; Van Sebille et al. 2015). The Arctic region has long been considered a pristine environment, relatively undisturbed by humans. However, recent studies have shown that plastic debris has reached the Arctic oceanic and sea-ice environments, and its wildlife (Schulz et al. 2010; Obbard et al. 2014; Lusher et al. 2015; Trevail et al. 2015b; Bergmann et al. 2017a, c; Buhl-Mortensen and Buhl-Mortensen 2017; Cózar et al. 2017). A suggested presence of an accumulation area in the Barents Sea (Van Sebille et al. 2015) was supported by Cózar et al. (2017) with recent field data and additional modelling indicating a peak accumulation of plastic in the vicinity of Svalbard and Novaya Zemlya. Microplastics were recorded from Arctic sea-ice cores (Obbard et al. 2014; Peeken in press) and in Arctic surface waters where levels of microplastic pollution fell in the range of those in other areas in the North Atlantic and the North Pacific (Lusher et al. 2015).

The ingestion of plastic debris by Arctic marine species has been recorded in organisms ranging from marine mammals (Martin and Clarke 1986; Finley 2001) to seabirds (Lydersen et al. 1989; Mallory 2008; Provencher et al. 2010; Trevail et al. 2015a) and blue mussels (*Mytilus* spp.; Lusher et al. 2017). However, to our knowledge there are hitherto only two reports of litter ingestion by an Arctic fish species, the Greenland shark (*Somniosus microcephalus*; Leclerc et al. 2012; Nielsen et al. 2013). The incidence of plastic ingestion in Arctic food webs is likely to increase as plastic pollution rises in the Arctic (Tekman et al. 2017). The ecological consequences of plastic ingestion are currently largely unknown. Large items may get stuck in organisms and obstruct the intestinal tract, and may cause injury or a false sense of satiation (Kühn et al. 2015); very small particles may translocate and pass to organs or cell with unknown consequences (Jani et al. 1992; Browne et al. 2008; Brennecke et al. 2016). Although not covered in this study, the recent detection of various persistent organic pollutants adsorbed to passive polyethylene samplers deployed west of Svalbard (Sun et al. 2016) highlights the potential of transfer of toxins upon ingestion by (Arctic) organisms (Tanaka et al. 2015; Chen et al. 2018).

Polar cod (*Boreogadus saida*) is regarded as a key species in the Arctic food web because it is regularly consumed by top predators (Lønne and Gabrielsen 1992; Mehlum and Gabrielsen 1993; Weslawski et al. 1994; Hop and Gjøsæter 2013) and because of its high energetic value (Hop and Gjøsæter 2013; David et al. 2015). They occur in large numbers directly underneath the Arctic sea ice (Lønne and Gulliksen 1989; Gradinger and Bluhm 2004; David et al. 2015). Young polar cod are strongly associated with the sea-ice habitat (Gradinger and Bluhm 2004; David et al. 2015; Kohlbach et al. 2017), as ice-associated amphipods and copepods are its main prey (Lønne and Gulliksen 1989; Kohlbach et al. 2017). In the under-ice layer, elevated levels of microplastics have been reported (Obbard et al. 2014; Peeken in press). The under-ice layer could thus be a zone of high plastic litter concentration and a source of plastics for organisms such as polar cod foraging in the under-ice environment. For this study, juvenile polar cod were investigated for the ingestion of plastics in order to provide a first baseline of marine plastic litter ingestion by a key fish species in the Arctic food web which forages specifically in the under-ice habitat.

## Methods

### Sampling

**Fig. 1:**
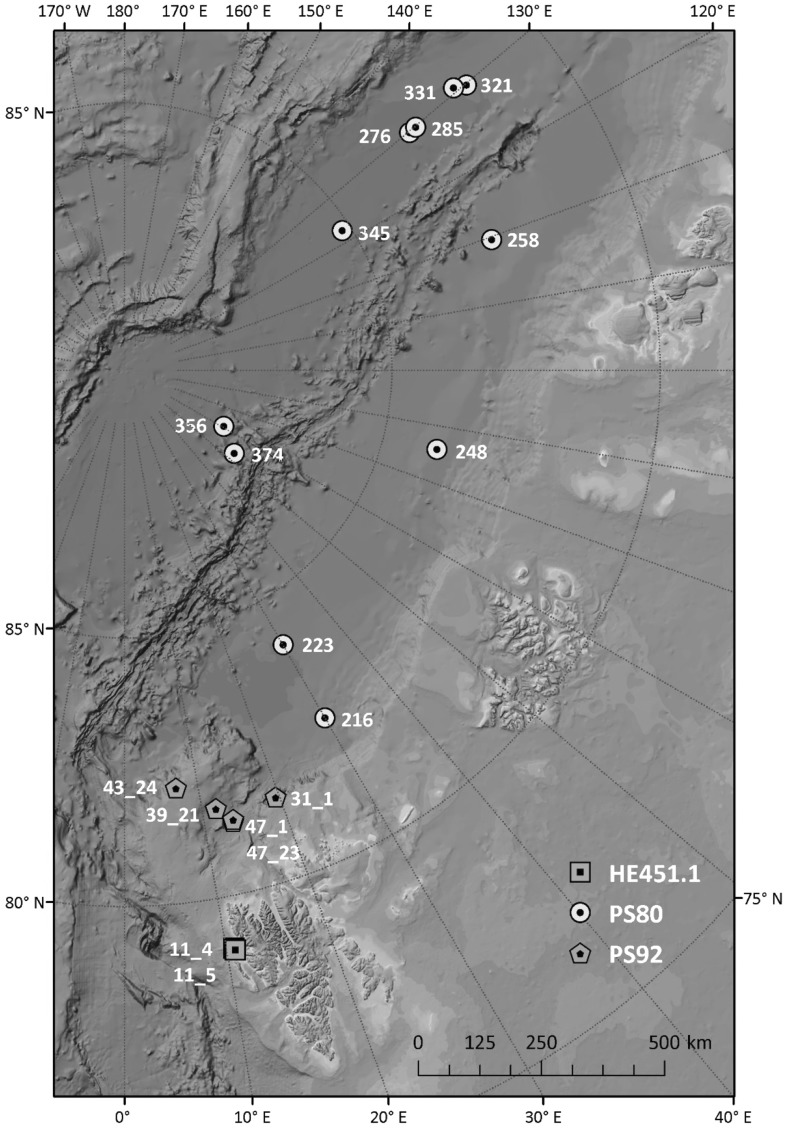
Map of sample stations for polar cod *(Boreogadus saida)* from three different research expeditions (HE451.1, squares; PS80, circles and PS92, pentagons). Numbers indicate stations where polar cod was caught (For details see Online Resource 2)

Samples of polar cod were collected during three research cruises in the Arctic between 2012 and 2015 (David et al. 2015; Mark 2015; Flores et al. 2016). Details of each cruise such as ship, date and fishing method are presented in Table 1. Sample locations are presented in Fig. 1. All individuals from PS80 and PS92 were collected in ice covered waters, while fish from the HE 451.1 expedition were caught in open water.

**Table 1:** Details on the three research cruises where polar cod (*Boreogadus saida*) used for stomach content analysis was collected

Expedition name	Expedition number	Research vessel	Area	Time	Fishing gear	Number of stations
IceARC	PS80	Polarstern	Eurasian basin	August–October 2012	SUIT	11
TRANSSIZ	PS92	Polarstern	Svalbard shelf, Yermak Plateau	June 2015	SUIT (+RMT 1 ind.)	5
	HE451.1	Heincke	Svalbard: Kongsfjorden, Billefjorden	September 2015	Juvenile fish trawl	2

During the expeditions PS80 and PS92 with the ice breaker RV *Polarstern*, fish were caught along the under-ice surface (0–2 m depth) using Surface and Under Ice Trawls (SUIT). SUIT was designed to sample the upper two metres of the water column either in open water or directly beneath the usually hardly accessible sea ice (Van Franeker et al. 2009; Flores et al. 2012). Half hour trawls were conducted at speeds between 2 and 3 knots. SUIT consists of a steel frame with a 2 × 2 m opening, and two nets with different mesh sizes attached (a 0.3 mm mesh and a 7 mm half-mesh). Floats attached to the frame ensure that the trawl stays at the surface or the sea-ice underside. A bridle connected to one side of the SUIT frame forces the trawl to shear out sideways, away from the wake of the ship, ensuring sampling underneath relatively undisturbed ice floes. A detailed description of SUIT sampling during PS80 is provided by David et al. (2015). One fish from PS92 was collected with a Rectangular Midwater Trawl (RMT) between 0 and 50 m depth.

Polar cod from open water near Svalbard was caught with a juvenile fish trawl at depths between 13 and 30 m towed at 2.7–3.3 km for 15 min at depths between 13 and 30 m. To effectively catch small and juvenile fish and surface them alive, a fish-lift (Holst and McDonald 2000) connected to the juvenile fish trawl was used.

### Stomach content analysis

In total, 72 individual polar cod were used for the purpose of this study. The total length, weight and sex of each individual were recorded. Although the initial reason for stomach analyses was an assessment of diet of the fishes, the samples were also used to assess plastic ingestion. The stomachs were dissected from the fish using scissors, which took place either on board or later in the laboratory. The stomach contents were removed from the stomachs by cutting them open and rinsing out the content into a clean petri dish using deionised water. All stomachs and extracted stomach contents were stored in 4% hexamine-buffered formaldehyde-sea water solution. In order to remove the formaldehyde solution, the stomach contents were rinsed for a few minutes by placing the sample on a 35 µm sieve under a running tap in a fume hood prior to the plastic analysis.

After carefully pouring the stomach content in a clean Bogorov counting chamber, samples were checked visually for plastics, using a Discovery V8 stereomicroscope (Zeiss, Germany). Suspect items were photographed and measured using an AxioCam MRc with AxioVision40 V 4.8.2.0 software (Zeiss, Germany) and collected for later analysis. All suspect particles except micro-fibres were analysed by µFTIR (Shimadzu FTIR IRTracer-100, Infrared Microscope AIM-9000, diamond cell (DC-3; Specac) to confirm whether a particle was of synthetic origin and to identify the polymer type. Spectra were measured in transmission mode on different points of the sample to avoid disturbance by surface fouling on the particles. Several reference libraries containing about 14,500 spectra in total were used to compare the detected spectra (Shimadzu Libraries, STJapan-Europe, standard data base from Biorad Sadtler and other libraries).

### Airborne fibre contamination

In this study and most other microplastic publications (e.g. Foekema et al. 2013; Rummel et al. 2016; Hermsen et al. 2017), the term ‘fibres’ refers to ‘micro-fibres’, the omnipresent dust-like bits from clothing, carpets or other woven garments. Micro-fibres in the marine environment are assumed to reach the oceans via sewage facilities (Browne et al. 2011) or atmospheric distribution (Dris et al. 2016). More sturdy ‘fibres’ that would not become airborne such as those derived from, e.g. multifilament ropes, network and fishing line, are addressed as ‘threadlike’ materials.

Secondary contamination of samples through airborne micro-fibre dust has been observed as a serious problem in earlier studies (e.g. Davison and Asch 2011; Foekema et al. 2013; Rummel et al. 2016). Wesch et al. (2017) even showed that such secondary fibre contamination is basically unavoidable in nearly any type of sampling and laboratory setting, and seriously affects results. For this reason, our primary goal was thus to quantify ingestion of non-fibrous plastic particles that are not subject to such airborne bias. Nonetheless, as the main type of plastics reported in the ice cores of Obbard et al. (2014) concerned micro-fibres and these micro-fibres are often used in studies to show a human impact on marine environments, we made all efforts to quantify and compare fibre abundance in our samples and controls.

The fish used for this diet study were, however, dissected without a specific protocol to avoid secondary pollution, and it is unclear how many airborne fibres might have polluted the samples before their processing in the plastic study. In spite of this caveat, the use of different handling protocols does provide an opportunity to investigate the effect of different aspects of the handling process on the number of fibres found in a sample.

From the combination of different field sampling methods and following analytical procedures, we arrived at five different protocols by which our samples had been handled (see Table 2). All the stomachs collected on Polarstern expedition PS80 (*n* = 49) were opened and rinsed out prior to the plastic investigations, but subsequent processing differed. In 19 cases (group A), the stomachs were opened and the natural diet of the polar cod was analysed (see Kohlbach et al. 2017), after which the analysed content was again preserved on formaldehyde-sea water solution.

**Table 2:** Overview of different method groups for the investigation of plastic ingestion by polar cod (*Boreogadus saida*)

Protocol group	Expedition	Number of samples	Stomach content extracted previously	Diet studied previously	Umbrella above sieve	Fibre control
A	PS80	19	Yes	Yes	No	No
B	PS80	13	Yes	No	No	No
C	PS80	17	Yes	No	Yes	No
D	PS92	9	No	No	No	Yes
E	HE 451.1	14	No	No	Yes	Yes

In the other cases (group B 13 individuals and C 17 individuals), the stomach contents were removed from the stomach and preserved directly on formaldehyde-sea water solution, without any prior analysis. The remaining 23 stomachs from the other expeditions (groups D and E) were preserved intact after the dissection of the fish, and opened with scissors directly before the plastic research was conducted. The stomachs that arrived unopened were rinsed with MilliQ from the outside. During the rinsing of the stomach contents of groups C and E (*n* = 31), a simple plastic sheet umbrella, connected to the tap, covered the sample in order to test if such addition could reduce potential secondary pollution by airborne fibres.

Scissors, tweezers, sieves and dishes were carefully rinsed with deionised water and inspected underneath the stereomicroscope before use. Precautions to prevent aerial fibre contamination were taken as far as possible by cleaning the workspace, wearing blue cotton lab coats, and as short as possible exposure of samples. Samples were covered with a clean glass lid whenever possible during processing and analysis. During microscopic analysis of samples in groups D and E (*n* = 23), a control petri dish filled with deionised water was placed next to the stereomicroscope and was checked after each sample of the previously unopened stomachs. No such controls were used in samples from groups A to C, because those stomachs had already been opened and processed to various extents before our investigations. No FTIR measurements were performed on the fibres.

A one-way ANOVA followed by a Tukey’s HSD post hoc test was performed to compare the number of fibres between the five different handling protocols applied. A non-parametric Wilcoxon Rank Sum test was used to evaluate the effect of an umbrella on fibre contamination during rinsing of samples between group B (no umbrella) and group C (umbrella), but showed no significant reduction of fibres in the sample. These two groups were chosen as both came from the same expedition and were further handled in the same way. The same test was used to investigate the effect of the stomach being previously opened (B and C) or not (D and E) on the amount of fibres in the sample.

For groups D and E, control petri dishes were placed next to the work space. With again a non-parametric Wilcoxon Sum Rank test, we tested whether the samples differed significantly from the controls and whether the controls differed between each other. The correlation between the number of fibres in a sample and the number of fibres in the corresponding control was tested using Pearson’s correlation coefficient, which ranges between − 1 and 1 with 0 indicating no correlation. The significance of found correlations was further tested by calculating a *t* value and corresponding *p* value based on Pearson’s product moment correlation coefficient. All statistical tests were conducted using R version 3.3.1. (R Core Team 2014).

## Results

The polar cod from PS80 used in this study had an average length of 78 mm (se 2.74), ranging from 52 to 137 mm. Their average weight was 3.49 g (se 0.49), ranging from 0.83 to 18.87 g. The fish from PS92 were larger with an average length of 107 mm (se 8.17), ranging from 63 to 157 mm. Their average weight was 9.58 g (se 1.65), ranging from 1.71 to 24.27 g. Fish caught during the HE451.1 expedition ranged from 44 to 62 mm in size, and had an average total length of 55 mm. Their weight ranged from 0.48 to 1.56 g, averaging at 1.01 g.

**Fig. 2:**
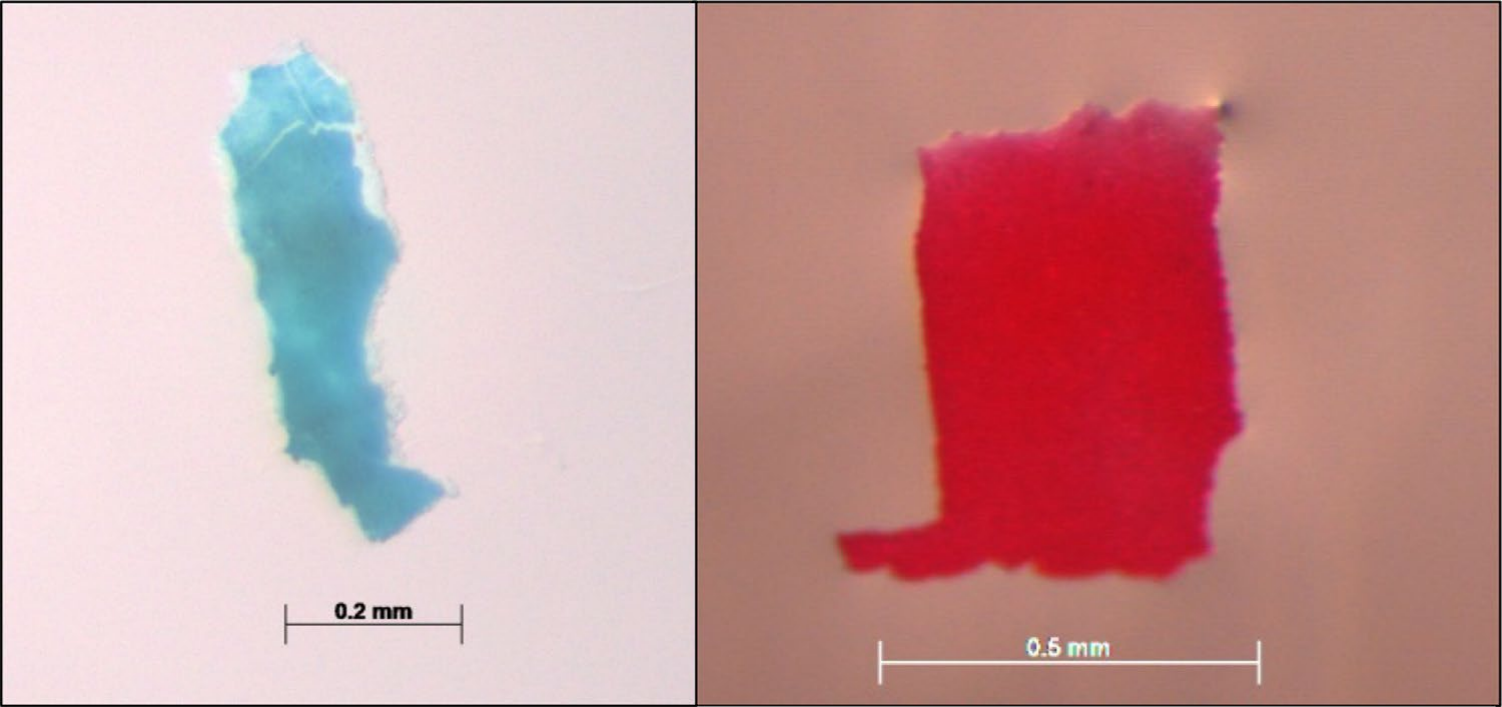
Photograph of microplastic found in stomachs of polar cod (*Boreogadus saida)*. Left: sheet HE451.1, fish P628; Right: sheet PS92, fish P590)

In total, 8 particles were collected that, from their combination of size, shape and/or colour, were suspected to be plastic. Fibres were excluded from this selection and discussed separately below because of the risk of representing secondary contamination. After µFTIR analysis, only two of these particles were confirmed to be synthetic polymers, originating from two different individuals from the expeditions HE451.1 and PS92, respectively (Fig. 2). Of the two fish that contained plastic, the first was a 93-mm-long male caught during PS92, and the other was a fish of 46 mm total length caught during HE451.1. The two plastic particles were identified as two sheets as they were both soft and flexible.

According to the µFTIR analysis, the red sheet most likely consisted of epoxy resins and had a size of 0.65 × 0.4 mm. The blue sheet had a kaolin base with embedded polymethylmethacrylate (PMMA) and had a size of 0.59 × 0.17 mm (both spectra and details on identification can be found in Online Resource 1). Both particles were thus in the microplastic size range (< 5 mm; Arthur et al. 2009; Thompson 2015).

Accordingly, we found non-fibrous microplastic particles in 0 out of 51 individuals from expedition PS80, as compared to 1 of 7 individuals from PS92 and 1 of 14 individuals from the expedition to Svalbard (HE451.1). This means that the overall frequency of occurrence of non-fibrous microplastic particles in 2 out of 72 polar cods equals 2.8% for the combined expeditions.

The other six particles that were suspected to be plastic initially were analysed with µFTIR as being cotton threads (*n* = 3) and protein (*n* = 3) and therefore these particles were not counted as plastics. Protein might originate from the fish diet such as crustacean shells. The analytical spectra are presented in Online Resource 1.

### Fibres

**Fig. 3:**
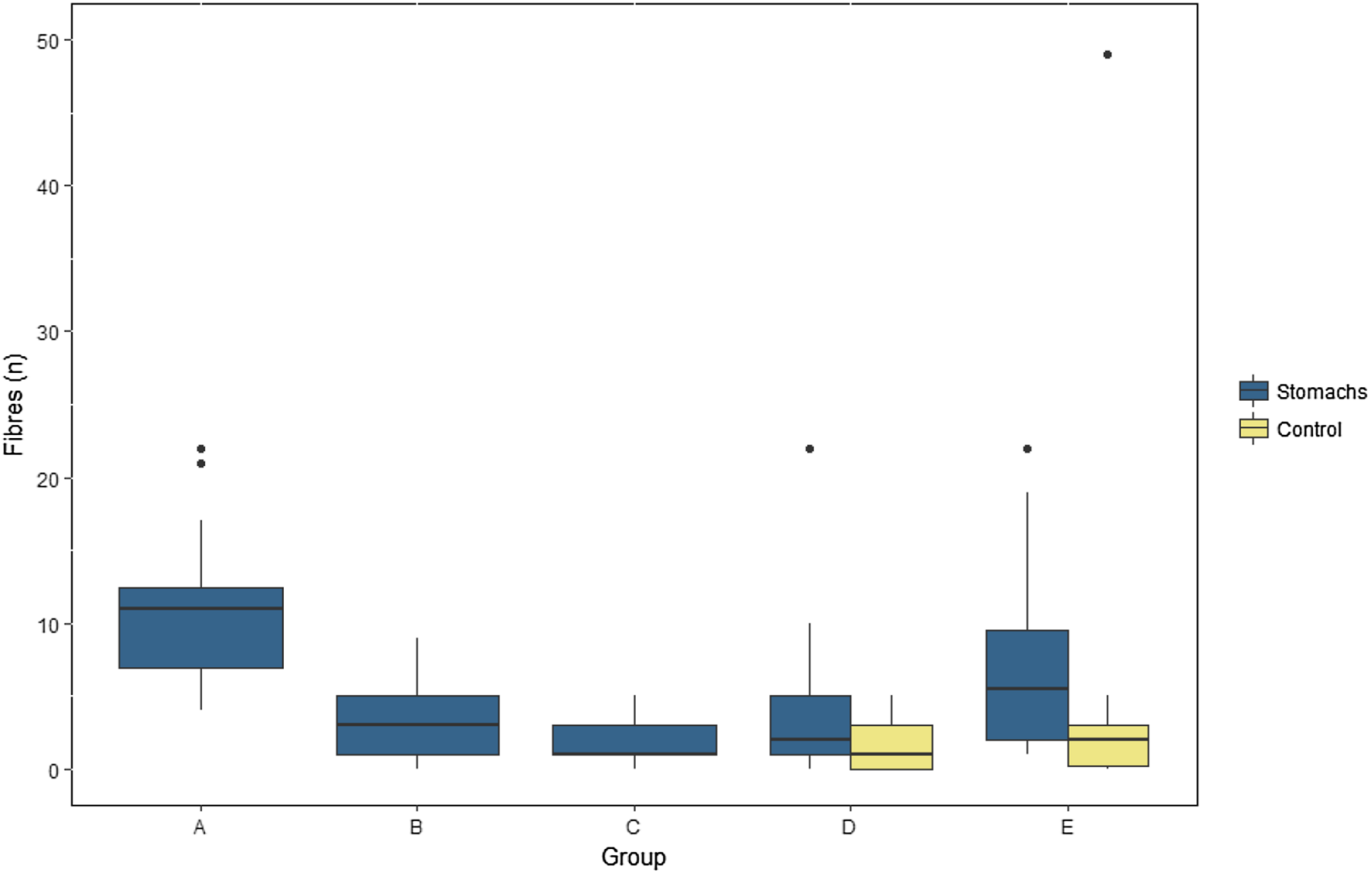
The number of fibres found in the stomachs of polar cod (*Boreogadus saida*, blue) according to the five different analysis protocols applied (group A–E; see Table 2 for details). Fibre controls are depicted in yellow (Groups D and E, right side). The horizontal black lines show the median number of fibres for all observations. The upper and lower limits of the coloured square boxes indicate the 25th and 75th percentile. The upper and lower limits of the vertical line indicate the minimum and maximum number of fibres in a group excluding the outliers (dots), which are numbers that are 1.5 times less or greater than the lower or upper percentiles, respectively. (Color figure online)

Micro-fibres were found in 90.2% of the samples, but in extremely variable quantities between and within the groups, as shown in Table 3 and Fig. 3. The number of fibres per fish stomach ranged between 0 and 22, and in controls between 0 and 49. The highest mean number of fibres was found in the stomachs of the group with the longest time exposed to the air without any specific protection measures used (Group A; Fig. 3).

**Table 3:** Average number of fibres recorded in the different sample groups of polar cod (*Boreogadus saida*) and in the control samples

Group	*n*	Average per sample ± SD	Average per control ± SD
A	19	10.9 ± 5.3	
B	13	3.1 ± 2.8	
C	17	2.3 ± 2.1	
D	9	5.2 ± 7.0	1.7 ± 1.9
E	14	7.3 ± 6.7	5.1 ± 12.7

The number of fibres in group A was significantly higher than in groups B, C and D (ANOVA *F*_4,67_ = 9.12, *p* < 0.00; Tukey’s HSD, *p* < 0.05). The number of fibres in the stomachs from group E was significantly higher than those in group C (ANOVA *F*_4,67_ = 9.12, *p* < 0.00; Tukey, *p* = 0.03). There was no significant difference in the number of fibres between the remaining groups (Groups B, C and D). The stomachs that had been opened before our plastic investigations did not have significantly more fibres per sample than the ones that were first opened during our own analysis. However, as samples came from different expeditions this result should be interpreted with caution.

For both groups D and E, the mean number of fibres was higher in the analysed stomachs than in the controls. However, the difference between the treatment and its corresponding control was not significant. There was also no significant difference in the number of fibres between both controls.

Although there was a match between the extremes in a sample and simultaneous control (22 fibres in the sample and 49 fibres in its control), linear regression and Pearson’s correlation (*t* = 2.028, df = 21, *p* > 0.05) revealed no significant overall correlation between the number of fibres in the samples and the number of fibres in the controls that could be used to create some sort of individual correction for the number of fibres in a sample based on the number of fibres in its control. Thus, our best estimate on the impact of aerial fibre contamination during laboratory analysis are the averages found in controls of groups D and E.

## Discussion

This first study of potential microplastic ingestion by polar cod sampled over a large part of the Central Arctic Ocean (CAO) and partly dwelling in the barely accessible under-ice habitat indicates that polar cod probably do ingest microplastics, albeit at very low frequencies. However, we have no comparison for our data with other aquatic organisms living within the sea-ice habitat. Our overall result of 2.8% frequency of occurrence of ingested non-fibrous microplastic particles among 72 polar cod is similar to the level of plastic ingestion observed in the full gastrointestinal tracts of Atlantic cod (*Gadus morhua*) from Newfoundland, where 2.4% of 205 fish analysed contained non-fibrous plastic (Liboiron et al. 2016). Prokhorova and Krivosheya (2013) reported two incidents in the Barents Sea of an Atlantic cod found to be entangled in fishing line and one individual with ingested plastic. By contrast, no plastic was detected in the stomachs of 114 Atlantic cod from northern Norway (Lofoten Islands and Varangerfjorden) after visual inspection of the stomach content under a microscope (Bråte et al. 2016).

Relatively high numbers of marine plastic debris have been noticed in Northern Fulmars from the vicinity of Svalbard. In this species, the larger picture of plastic ingestion shows decreases with higher latitude, probably related to the distance to highly urbanised areas (Kühn and Van Franeker 2012). However, within that trend, Trevail et al. (2015a) found a slightly elevated incidence of plastic ingestion in Northern Fulmars around Svalbard and suggested a potential relation with a sixth accumulation area as modelled by Van Sebille et al. (2015) and Cózar et al. (2017). Unfortunately, both the fulmar data and our polar cod data do not have the spatial resolution to evaluate further details of the Cózar et al. (2017) model.

Even though the field of microplastic research is maturing, there are still examples of recent research where fibres are considered as anthropogenic debris ingested from the environment, where no controls on airborne fibres are conducted (or reported) and potential sources of secondary pollution are neglected (e.g. Steer et al. 2017). Based on the controls conducted during our analyses, and the poorly controlled conditions during earlier steps in the collection of our samples, we have no reason to assume that synthetic fibres in our samples were derived from ingestion by the fish, as most fibres can be explained in terms of secondary contamination. If the average numbers of fibres found in the controls for groups D and E (1.7 and 5.1 fibres, respectively) are assumed to have been similar during our analyses of stomachs in groups B and C (3.1 and 2.3 fibres per stomach), the number of fibres found ingested by the fish would be negligible. The much higher number of fibres in group A, which has the same source as groups B and C, can be explained by exposure during the earlier diet study and second time of storage without special precautions against aerial fibre contamination. Accordingly, our analysis could not doubtlessly quantify ingested fibres, even if they may have well been present in the stomachs. This illustrates that it should be kept in mind that the controls for aerial contamination only cover the phase of our microscopic analysis for plastics, and that it remains unknown how much contamination occurred during the dissection of fish and stomachs that have been opened prior to the plastic analysis. The variation found in the numbers of fibres in the stomach contents as well as the controls suggest that there are many factors influencing the rate of pollution, including factors such as number of people present in the lab and their behaviour. As a consequence, it was not possible to assess whether polar cod were affected to a major extent by the fibres reported from sea ice (Obbard et al. 2014) and in open Arctic seawater (Lusher et al. 2015).

When studying microplastic ingestion by marine organisms, a protocol should be established that takes proper account of secondary pollution (see e.g. Torre et al. 2016; Hermsen et al. 2017; Wesch et al. 2017) in order to avoid bias in estimates of impacts of anthropogenic waste to marine organisms.

The umbrella that was tested during sample rinsing under the fume hood did not lead to a significant reduction of fibres in the samples. This suggests that rinsing was not a major contributor to airborne fibre contamination under the given circumstances. However, potential addition of fibres that might occur in tap water, as demonstrated by Kosuth et al. (2017), could not be excluded. As most of the fibres encountered in the current study were supposed to be secondary pollution, no FTIR analysis was performed. FTIR analysis would probably help to identify fibres from clothing in contrast to threadlike material derived from fishing gear due to different polymer types used. In data presentations, splitting study results in separate categories of micro-fibres, as opposed to non-fibrous plastic particles unlikely to be spread by air, will be an essential component for the future. Although at low frequency, our results do confirm that anthropogenic waste, particularly plastic debris, has not only reached pristine Arctic regions but can also be found in marine organisms closely related to the sea-ice environment. The low frequency of plastic ingestion observed in polar cod suggests that at present life dwelling under Arctic sea ice may be relatively unaffected by anthropogenic plastic pollution.

Regardless of the exact ecological consequences, plastic contamination of Arctic ecosystems and its biota likely exerts further pressure on a system that is already suffering from the impacts of global change (Wassmann et al. 2011). In the future, increased influx of Atlantic water and accelerated ice drift (Spreen et al. 2011; Walczowski et al. 2012) may enhance the advection of microplastic particles into the CAO, and hence their potential as an environmental stressor for polar cod. To better assess the regional variability of plastic ingestion by the ecological key species polar cod and potential changes over time with more certainty, we recommend geographically distributed and repeated studies. These studies should account rigorously for avoidable sources of secondary pollution, based on the experiences with this and other publications.

## Electronic supplementary material

Below is the link to the electronic supplementary material.

Supplementary material 1 (DOCX 1165 kb)
