# Supplementary material for: Plastic ingestion by juvenile polar cod (Boreogadus saida) in the Arctic Ocean
Source: Polar Biol. 2018 Feb 20;41(6):1269–78. doi: 10.1007/s00300-018-2283-8 (PMC6445488; doi:10.1007/s00300-018-2283-8)
Supplement: Supplementary file 1 — Supplementary material 1 (DOCX 1165 kb) [file 300_2018_2283_MOESM1_ESM.docx]

**Plastic ingestion by polar cod (*Boreogadus saida*) in the Arctic Ocean**

Susanne Kühn^1^, Fokje L. Schaafsma^1^, Bernike van Werven², Hauke Flores³, Melanie Bergmann³, Marion Egelkraut-Holtus^4^, Mine B. Tekman³, Jan A. van Franeker^1^

^1^Wageningen Marine Research, Ankerpark 27, 1781 AG, Den Helder, The Netherlands

²University of Utrecht, Heidelberglaan 2, 3584 CS Utrecht, The Netherlands

³Alfred-Wegener-Institut, Helmholtz-Zentrum für Polar- und Meeresforschung, Am Handelshafen 12, 27570 Bremerhaven, Germany

^4^Shimadzu Europa GmbH, Albert-Hahn-Str. 6-10, 47269 Duisburg, Germany

**Online Resource**

Contents

[Online Resource 1. Spectra of all suspect items 2](#_Toc504728613)

[Online Resource 2. Table of polar cod with plastic & fibre details 11](#_Toc504728614)

## Online Resource 1. Spectra of all suspect items

Suspect items retrieved from stomachs of polar cod were analysed with µFTIR (Shimadzu FTIR IRTracer-100, Infrared Microscope AIM-9000, diamond cell (DC-3; Specac), spectra were measured in transmission mode) to confirm whether it was anthropogenic debris and to possibly identify the polymer type. Several libraries with in total about 14500 spectra were used to compare the detected spectra (Shimadzu Libraries, STJapan-Europe, standard data base from Biorad Sadtler and other libraries). In total 8 particles were collected that from their combination of size, shape and/or colour, were visually suspected to be plastic. Fibres were excluded from this selection, because of the risk of representing secondary contamination. After µFTIR analysis only two of these pieces were confirmed to be plastic, originating from two different individuals. Of the 6 particles that were not confirmed to be plastics, the spectra are presented with alternative suggestions of their characteristics.

**Sample HE 451.1 P628 Plastic**


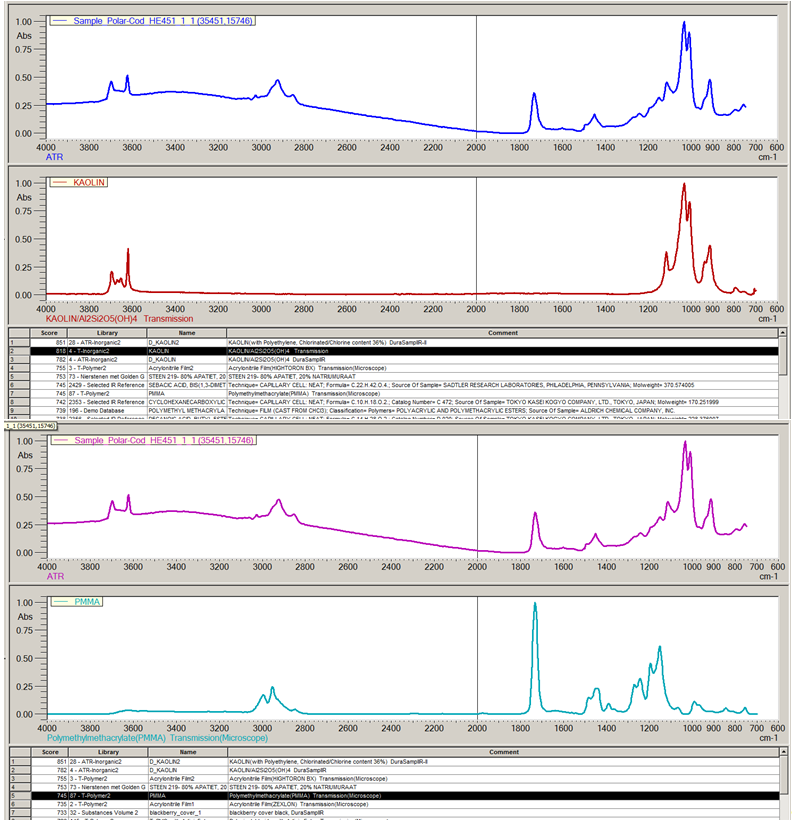


**Screenshot showing Kaolin as first match with a hit quality of over 800 (first scan). The centre scan represent the sample. The polar cod particle spectrum is a mixture of two substance from inorganic and organic source. PMMA with a hit quality of 745 (last scan). Both are inside the particle and only partly overlay by superposition. After subtraction of PMMA spectrum from the raw data the search was repeated. The match was once again Kaolin for the remaining signal groups.**

**Sample PS 92 P590 Plastic**


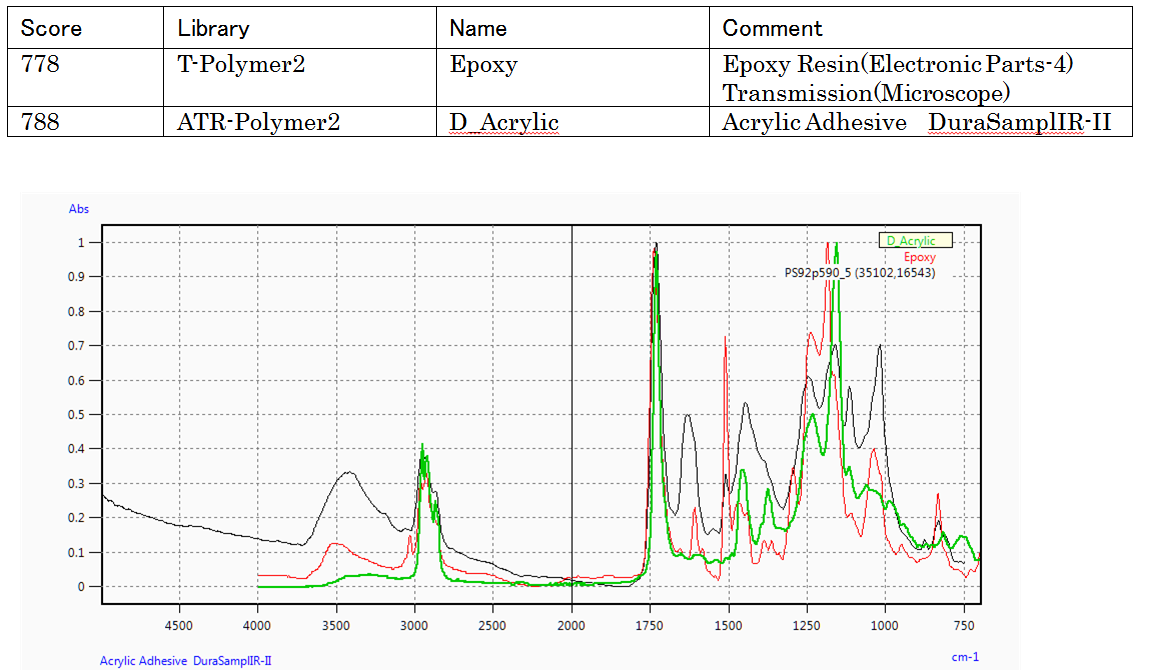


**The black spectrum is the sample measured, green represents an acrylic adhesive (Hit 788) and the red line shows the spectrum of epoxy (Hit 778).**


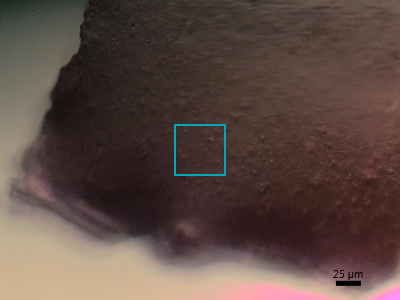


**Detailed picture of the plastic particle under µFTIR microscope. The square frame indicated the position of one of the repetitive µFTIR measurement.**

| Score | Library | Name | Comment |
| --- | --- | --- | --- |
| 880 | IRs Polymer2 | SKIN | Human skinATR/Diamond ATRcorrected |
| 877 | T-Polymer2 | Human Hair | Protein(Human Hair) Transmission(Microscope) |
| 872 | T-Polymer2 | Soy Bean Powder | Protein(Soy Bean Powder) Transmission(Microscope) |

**Sample PS 80 P106 – No plastic**


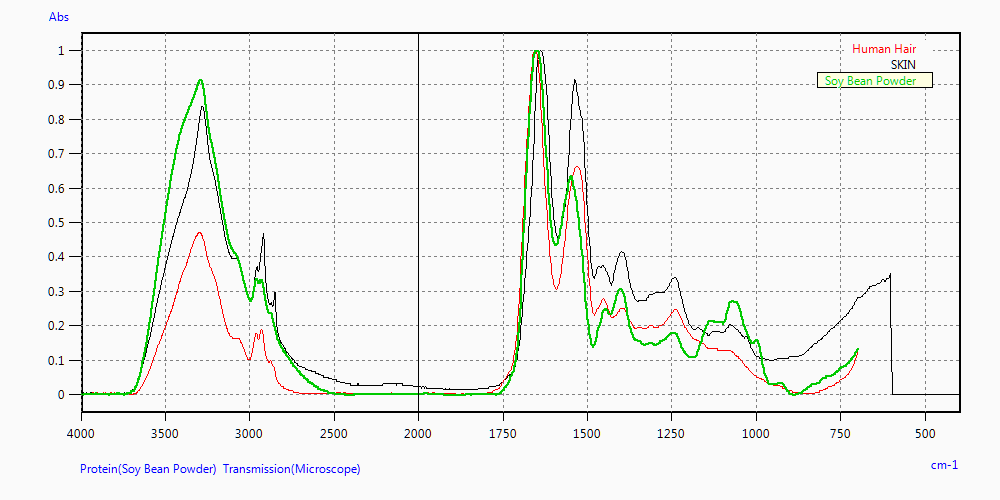


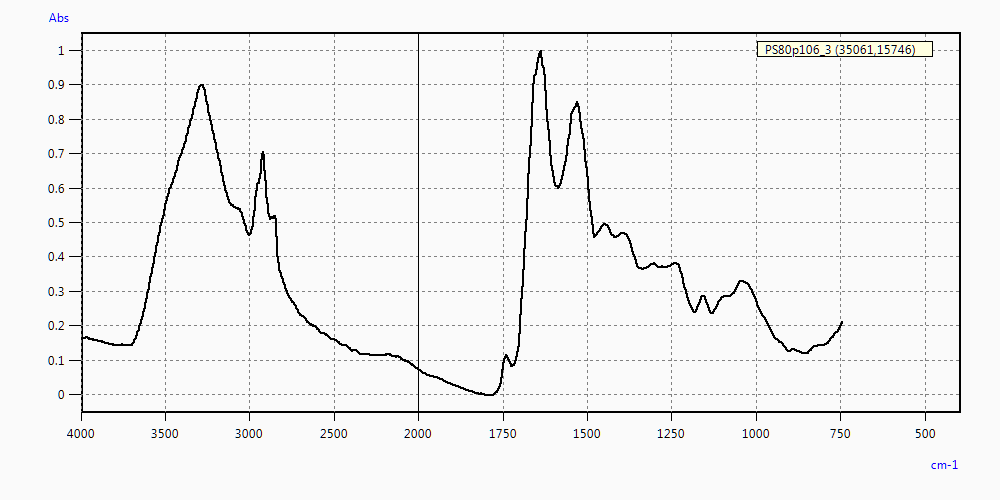


**The table shows the three most likely hits for the spectrum measured for that particle. The upper graph indicates these three hits, being either skin/hair (black line, Hit 880), human hair/protein (red line, Hit 877) and Soy bean protein (green line, Hit 872). The Bottom graph is the spectrum of the measured sample (black line).**

**PS80 P179 – No plastic**

| Score | Library | Name | Comment |
| --- | --- | --- | --- |
| 844 | T-Polymer2 | Soy Bean Powder | Protein(Soy Bean Powder) Transmission(Microscope) |
| 815 | IRs Polymer2 | SKIN | Human skinATR/Diamond ATRcorrected |
| 799 | ATR-Polymer2 | D_Protein2 | Protein(Soy Bean Powder) DuraSamplIR-II |


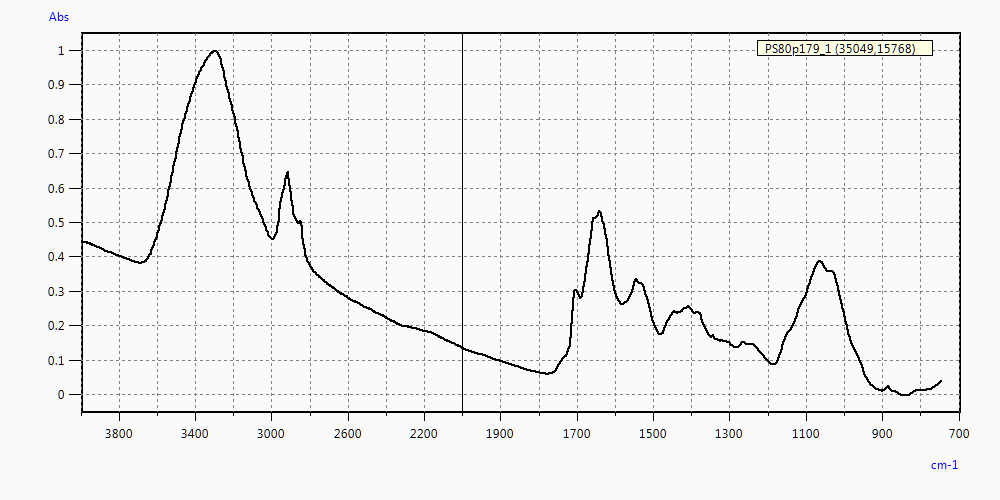

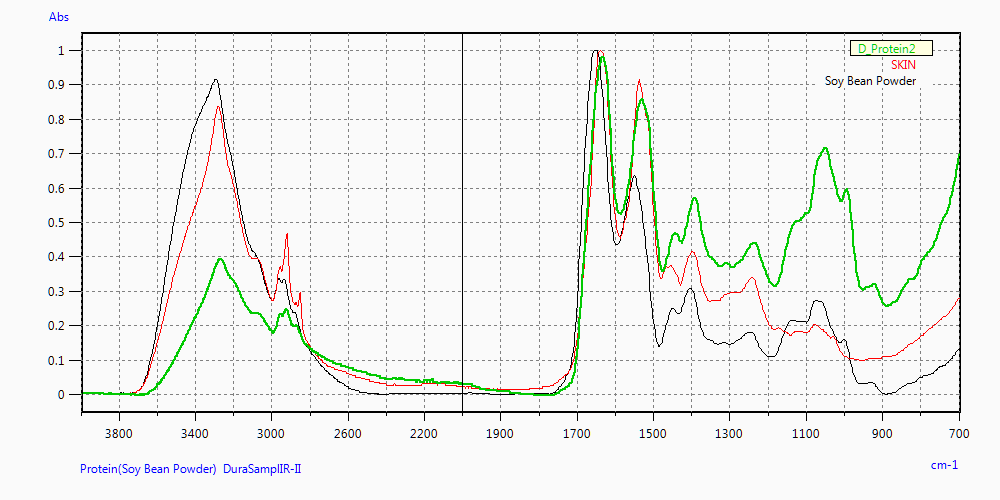
 **The table shows the three most likely hits for the spectrum measured for that particle. The upper graph indicates these three hits, being either Soy bean powder (black line, Hit 844), skin (red line, Hit 815) and protein (green line, Hit 799). The Bottom graph is the spectrum of the measured sample (black line).**

**PS80 P550**

| Score | Library | Name | Comment |
| --- | --- | --- | --- |
| 939 | T-Polymer2 | Cotton | Cotton Transmission(Microscope) |


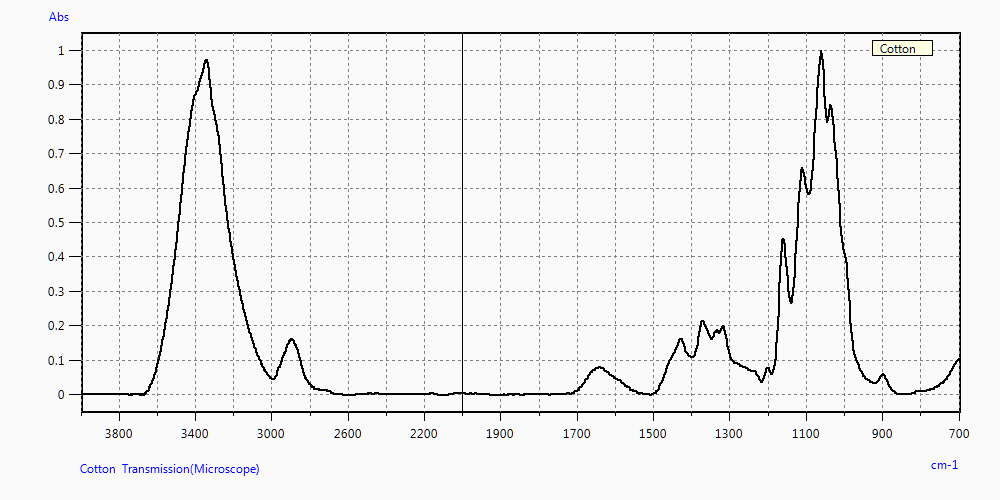


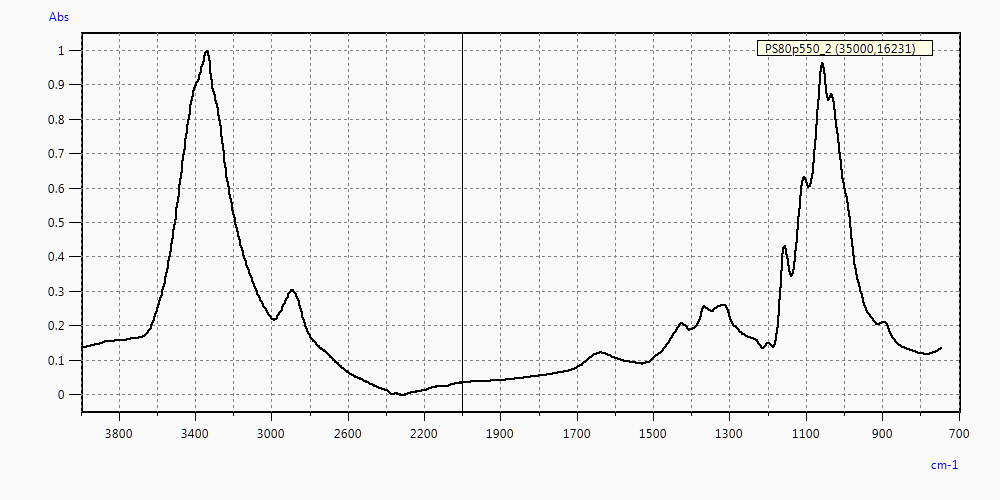


**The table shows the most likely hit for the spectrum measured for that particle. The upper graph clearly indicates cotton (Hit 939). The Bottom graph is the spectrum of the measured sample.**

**PS 80 P540 – No plastic**

| Score | Library | Name | Comment |
| --- | --- | --- | --- |
| 771 | IRs Polymer2 | COTTON | Cotton FiberATR/diamond ATRcorrected |


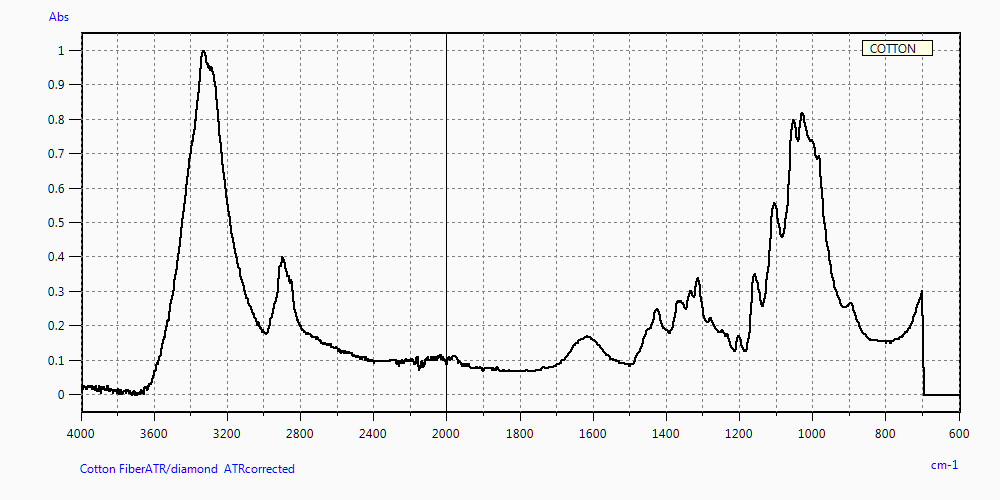

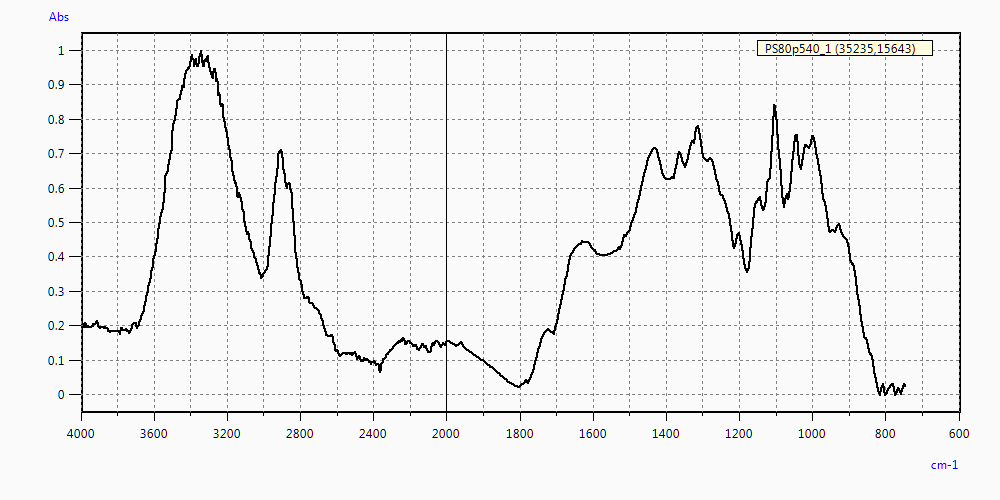


**The table shows the most likely hit for the spectrum measured for that particle. The upper graph indicates cotton (Hit 771). The bottom graph is the spectrum of the measured sample.**

**PS92 P605 – No plastic**

| Score | Library | Name | Comment |
| --- | --- | --- | --- |
| 790 | T-Polymer2 | Ramie | Ramie Transmission(Microscope) |


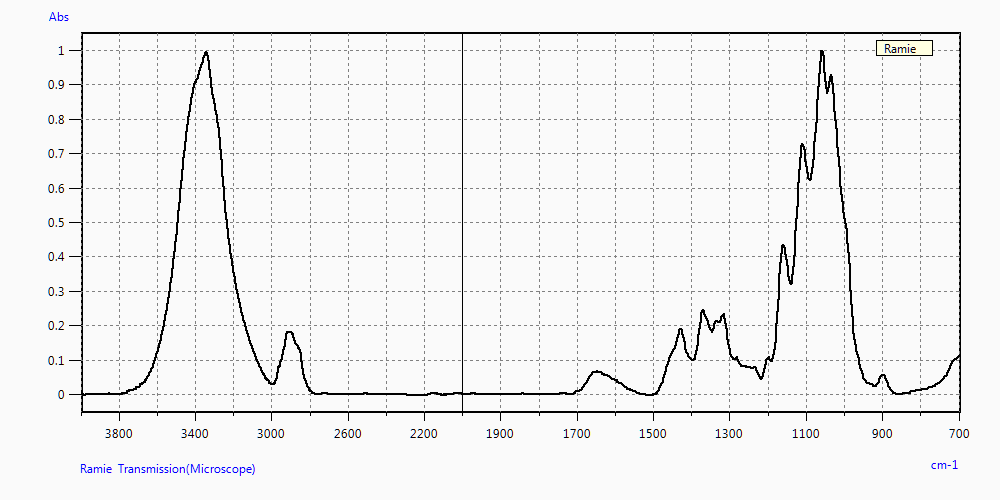

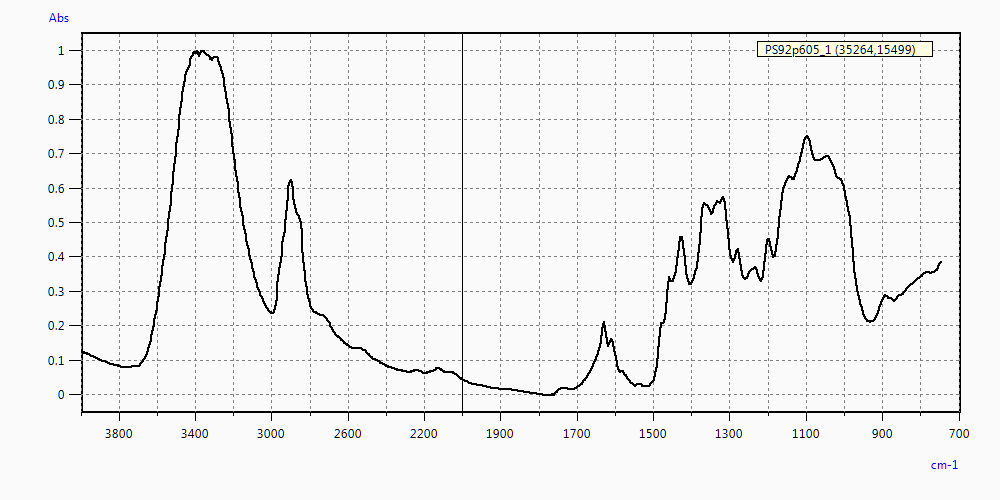


**The table shows the most likely hit for the spectrum measured for that particle. The upper graph indicates ramie, a type of cotton (Hit 790). The Bottom graph is the spectrum of the measured sample.**

**HE451.1 P610 No plastic**


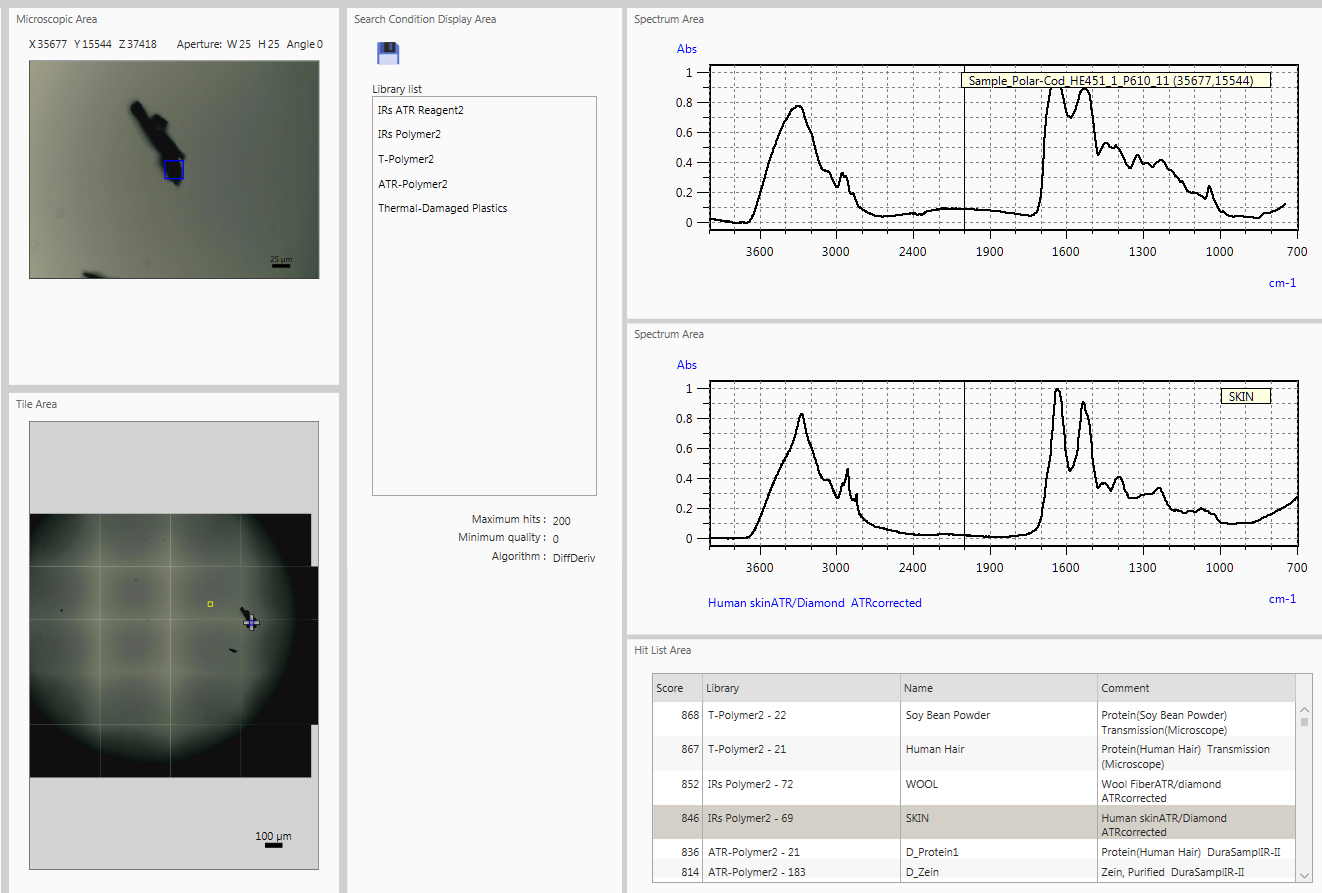


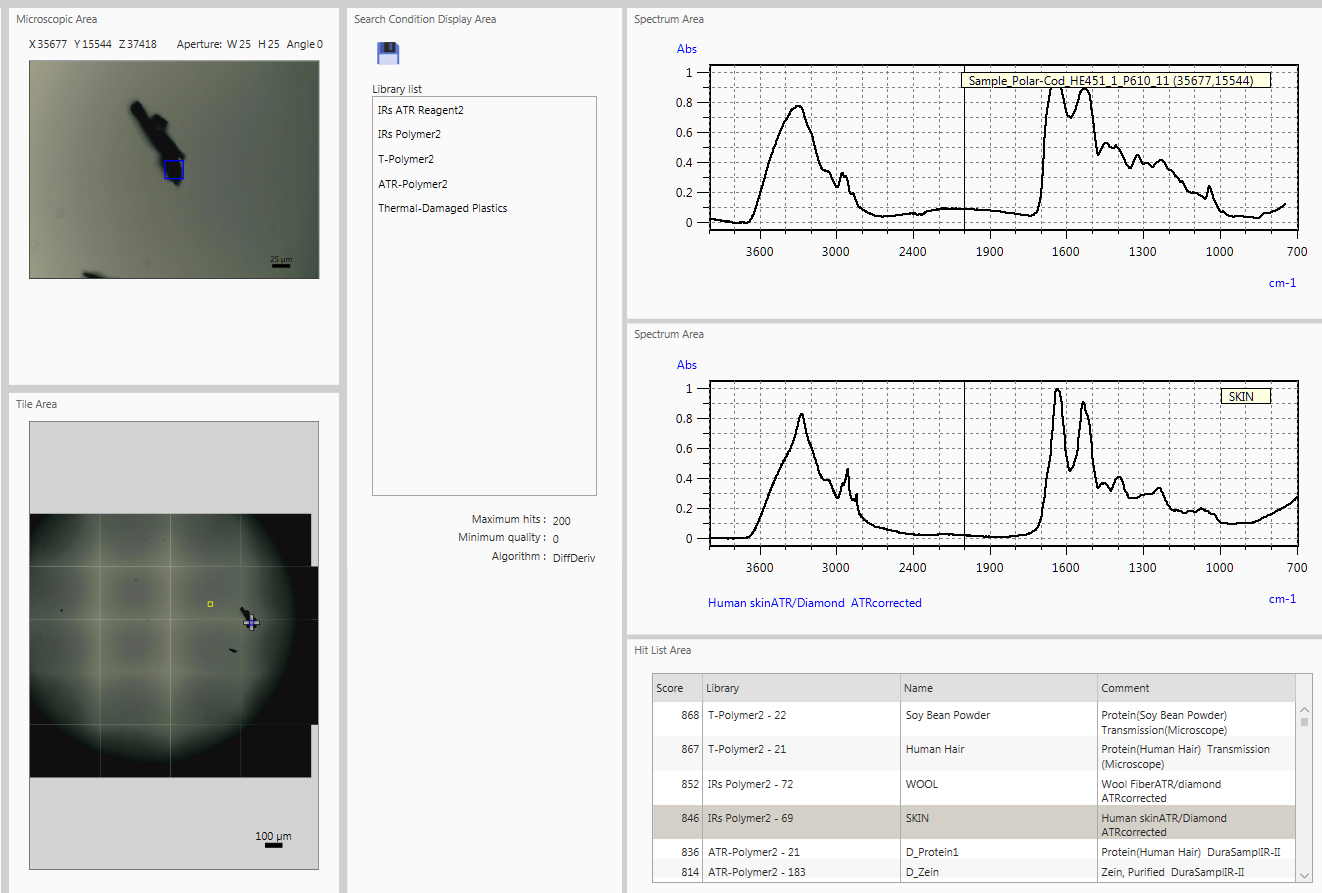


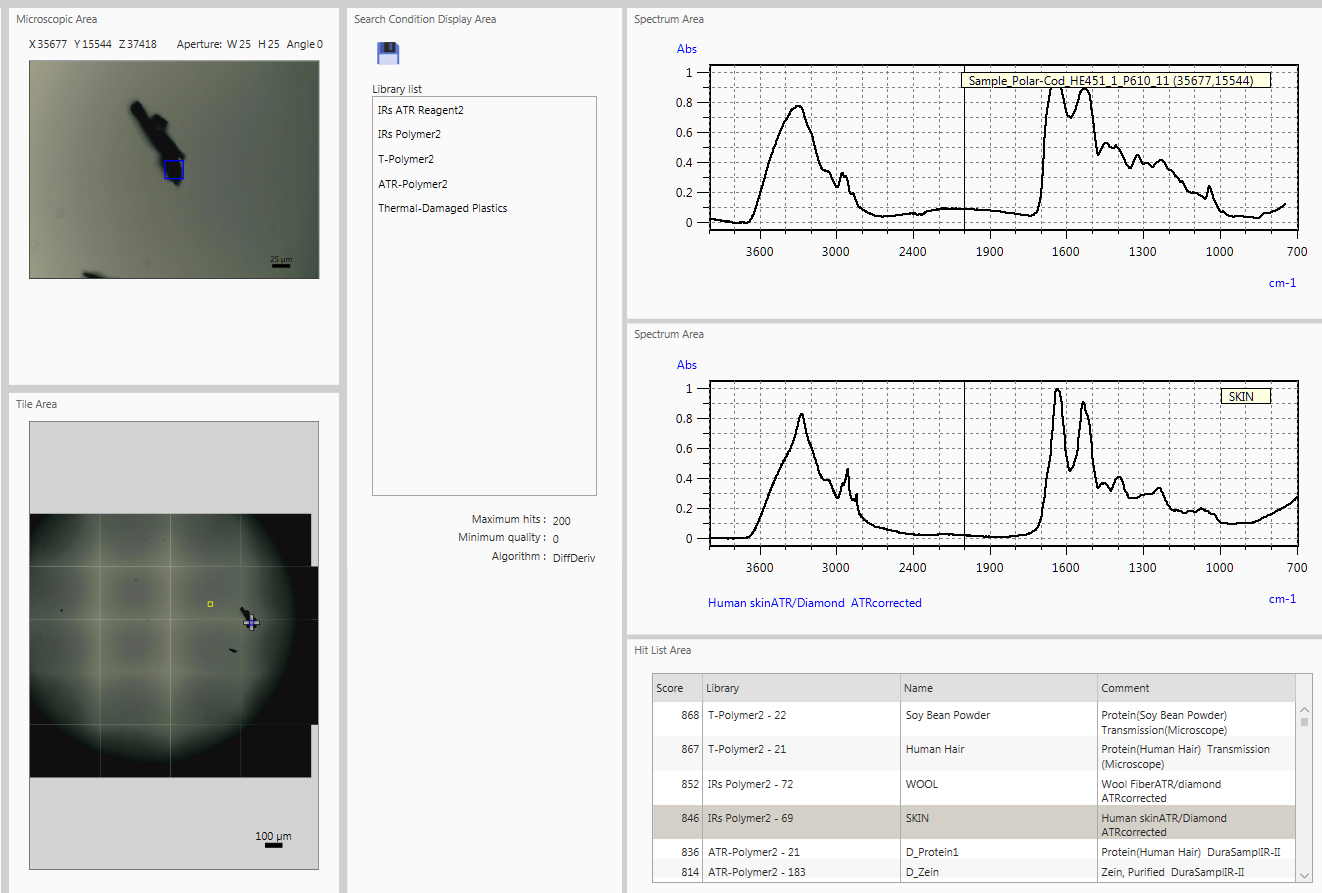


**The table shows the most likely hit for the spectrum measured for that particle. The upper graph indicates Skin, as after subtracting water from the spectrum skin seems to be the most likely substance (Hit 790). The Bottom graph is the spectrum of the measured sample.**

## Online Resource 2. Table of polar cod with plastic & fibre details

Table 1. Details of measurements, handling protocol, plastic and fibres detected per polar cod

| **Protocol group** | **Expedition** | **Fish (P)** | **Station** | **Length (mm)** | **Weight (g)** | **Sex (M=male, F=female, U=unknown)** | **Stomach opened before?** | **Diet studied before (AWI)** | **Umbrella applied above sieve** | **Number of non-fibrous plastic** | **Number of fibres in stomach sample** | **Fibre control** |
| --- | --- | --- | --- | --- | --- | --- | --- | --- | --- | --- | --- | --- |
| **A** | PS80 | 106 | 345 | 58 | 1.0 | M | yes | yes | no | 0 | 11 | n/a |
|  | PS80 | 134 | 285 | 84 | 3.6 | M | yes | yes | no | 0 | 7 | n/a |
|  | PS80 | 179 | 321 | 66 | 1.9 | M | yes | yes | no | 0 | 17 | n/a |
|  | PS80 | 183 | 216 | 76 | 2.6 | F | yes | yes | no | 0 | 6 | n/a |
|  | PS80 | 200 | 216 | 137 | 18.9 | M | yes | yes | no | 0 | 22 | n/a |
|  | PS80 | 250 | 374 | 89 | 5.2 | M | yes | yes | no | 0 | 10 | n/a |
|  | PS80 | 103 | 345 | 71 | 2.2 | M | yes | yes | no | 0 | 12 | n/a |
|  | PS80 | 226 | 345 | 62 | 1.3 | U | yes | yes | no | 0 | 13 | n/a |
|  | PS80 | 192 | 216 | 85 | 3.6 | M | yes | yes | no | 0 | 4 | n/a |
|  | PS80 | 327 | 276 | 58 | 1.2 | M | yes | yes | no | 0 | 4 | n/a |
|  | PS80 | 211 | 223 | 86 |  | F | yes | yes | no | 0 | 12 | n/a |
|  | PS80 | 121 | 331 | 59 | 1.2 | M | yes | yes | no | 0 | 7 | n/a |
|  | PS80 | 229 | 345 | 62 | 1.4 | M | yes | yes | no | 0 | 11 | n/a |
|  | PS80 | 136 | 285 | 92 | 4.3 | M | yes | yes | no | 0 | 16 | n/a |
|  | PS80 | 143 | 223 | 102 | 6.2 | M | yes | yes | no | 0 | 10 | n/a |
|  | PS80 | 110 | 345 | 70 | 2.3 | F | yes | yes | no | 0 | 4 | n/a |
|  | PS80 | 118 | 345 | 110 | 8.6 | M | yes | yes | no | 0 | 21 | n/a |
|  | PS80 | 233 | 345 | 73 | 2.9 | M | yes | yes | no | 0 | 9 | n/a |
|  | PS80 | 139 | 223 | 90 | 4.8 | M | yes | yes | no | 0 | 11 | n/a |
| **B** | PS80 | 198 | 216 | 94 | 4.5 | M | yes | no | no | 0 | 5 | n/a |
|  | PS80 | 540 | 285 | 55 | 1.0 | M | yes | no | no | 0 | 0 | n/a |
|  | PS80 | 550 | 285 | 71 | 2.8 | M | yes | no | no | 0 | 9 | n/a |
|  | PS80 | 525 | 216 | 108 | 6.1 | M | yes | no | no | 0 | 4 | n/a |
|  | PS80 | 528 | 216 | 100 | 6.4 | M | yes | no | no | 0 | 2 | n/a |
|  | PS80 | 517 | 216 | 90 | 4.5 | M | yes | no | no | 0 | 1 | n/a |
|  | PS80 | 203 | 248 | 76 |  | M | yes | no | no | 0 | 0 | n/a |
|  | PS80 | 217 | 356 | 77 | 2.7 | M | yes | no | no | 0 | 0 | n/a |
|  | PS80 | 221 | 356 | 80 | 3.4 | M | yes | no | no | 0 | 1 | n/a |
|  | PS80 | 246 | 374 | 89 |  | U | yes | no | no | 0 | 4 | n/a |
|  | PS80 | 171 | 321 | 54 | 0.9 | M | yes | no | no | 0 | 3 | n/a |
|  | PS80 | 253 | 258 | 56 | 1.2 | M | yes | no | no | 0 | 6 | n/a |
|  | PS80 | 168 | 321 | 53 | 0.8 | M | yes | no | no | 0 | 5 | n/a |
| **C** | PS80 | 237 | 345 | 81 | 3.8 | M | yes | no | yes | 0 | 2 | n/a |
|  | PS80 | 154 | 321 | 111 |  | M | yes | no | yes | 0 | 0 | n/a |
|  | PS80 | 265 | 258 | 64 | 1.9 | M | yes | no | yes | 0 | 1 | n/a |
|  | PS80 | 308 | 276 | 67 | 2.1 | M | yes | no | yes | 0 | 1 | n/a |
|  | PS80 | 355 | 276 | 62 | 1.4 | M | yes | no | yes | 0 | 5 | n/a |
|  | PS80 | 148 | 321 | 75 | 3.8 | M | yes | no | yes | 0 | 4 | n/a |
|  | PS80 | 345 | 276 | 77 | 2.8 | M | yes | no | yes | 0 | 0 | n/a |
|  | PS80 | 272 | 258 | 93 | 5.7 | M | yes | no | yes | 0 | 1 | n/a |
|  | PS80 | 333 | 276 | 58 | 1.2 | M | yes | no | yes | 0 | 0 | n/a |
|  | PS80 | 151 | 321 | 67 |  | M | yes | no | yes | 0 | 3 | n/a |
|  | PS80 | 360 | 276 | 84 | 2.6 | M | yes | no | yes | 0 | 1 | n/a |
|  | PS80 | 349 | 276 | 52 | 0.8 | M | yes | no | yes | 0 | 2 | n/a |
|  | PS80 | 150 | 321 | 64 |  | M | yes | no | yes | 0 | 3 | n/a |
|  | PS80 | 299 | 276 | 69 | 2.0 | M | yes | no | yes | 0 | 4 | n/a |
|  | PS80 | 279 | 258 | 105 |  | F | yes | no | yes | 0 | 4 | n/a |
|  | PS80 | 141 | 223 | 118 | 9.2 | M | yes | no | yes | 0 | 1 | n/a |
|  | PS80 | 304 | 276 | 69 | 2.0 | M | yes | no | yes | 0 | 1 | n/a |
| **D** | PS92 | 590 | 31_1 | 93 | 5.4 | M | no | no | no | 1 | 1 | 0 |
|  | PS92 | 605 | 31_1 | 122 | 11.9 | U | no | no | no | 0 | 4 | 0 |
|  | PS92 | 559 | 39_21 | 105 | 6.4 | F | no | no | no | 0 | 5 | 1 |
|  | PS92 | 573 | 43_24 | 63 | 1.7 | U | no | no | no | 0 | 10 | 3 |
|  | PS92 | 578 | 47_1 | 75 | 2.9 | F | no | no | no | 0 | 2 | 4 |
|  | PS92 | 575 | 47_23 | 127 | 15.3 | F | no | no | no | 0 | 22 | 5 |
|  | PS92 | 588 | 31_1 | 118 | 10.0 | M | no | no | no | 0 | 1 | 0 |
|  | PS80* | 242 | 374 | 80 | 3.8 | M | no | no | no | 0 | 2 | 1 |
|  | PS80* | 207 | 248 | 68 | 2.3 | M | no | no | no | 0 | 0 | 1 |
| **E** | HE 451.1 | 599 | 15-05 | 62 | 1.6 | U | no | no | yes | 0 | 22 | 49 |
|  | HE 451.1 | 619 | 11-04 | 62 | 1.4 | U | no | no | yes | 0 | 3 | 5 |
|  | HE 451.1 | 616 | 11-04 | 60 | 1.3 | U | no | no | yes | 0 | 19 | 2 |
|  | HE 451.1 | 612 | 15-05 | 44 | 0.5 | U | no | no | yes | 0 | 10 | 3 |
|  | HE 451.1 | 610 | 15-05 | 51 | 0.8 | U | no | no | yes | 0 | 4 | 4 |
|  | HE 451.1 | 628 | 11-04 | 46 | 0.6 | U | no | no | yes | 1 | 7 | 3 |
|  | HE 451.1 | 607 | 15-05 | 60 | 1.3 | U | no | no | yes | 0 | 1 | 2 |
|  | HE 451.1 | 601 | 15-05 | 58 | 1.3 | U | no | no | yes | 0 | 2 | 1 |
|  | HE 451.1 | 625 | 11-04 | 51 | 0.8 | U | no | no | yes | 0 | 2 | 0 |
|  | HE 451.1 | 622 | 11-04 | 56 | 1.2 | U | no | no | yes | 0 | 1 | 0 |
|  | HE 451.1 | 593 |  |  |  | U | no | no | yes | 0 | 2 | 0 |
|  | HE 451.1 | 631 |  |  |  | U | no | no | yes | 0 | 13 | 0 |
|  | HE 451.1 | 634 |  |  |  | U | no | no | yes | 0 | 8 | 2 |
|  | HE 451.1 | 637 |  |  |  | U | no | no | yes | 0 | 8 | 1 |

* Two samples from PS 80 were moved to group D as the handling protocol differed.
